# Psychophysiological markers of trust in automation: insights from ERP responses in a modified flanker task

**DOI:** 10.1186/s41235-026-00716-y

**Published:** 2026-03-25

**Authors:** Mallory C. Stites, Laura E. Matzen, Breannan C. Howell, Danielle S. Dickson

**Affiliations:** Sandia National Laboratories, PO Box 5800, Albuquerque, NM 87185 USA

**Keywords:** Trust in automation, ERPs, Decision-making, Human–machine teaming, ERN, P300

## Abstract

This study investigated the sensitivity of event-related potentials (ERP) to factors influencing trust in machine learning (ML) automation, specifically ML reliability, bias, and transparency, with the goal of identifying an electrophysiological marker of trust in automation. Participants performed a flanker task and observed a simulated ML algorithm perform a modified flanker task, while ERP data were collected. The performance flanker task showed canonical patterns in behavioral responses, including fewer errors and shorter response times to congruent trials. We also observed the expected ERP components, including the error-related negativity (ERN) and positivity (Pe), alongside a significant late positive component (LPC) associated with error processing. Contrary to predictions, no differences in oERN amplitudes were observed across model error conditions. The oPe component was elicited by model errors, yet was insensitive to model reliability or bias. Notably, an LPC was also observed to model errors and was larger for errors from the more reliable model (90% vs. 60%). LPC amplitude was negatively correlated with subjective trust ratings in the 60% reliable biased condition, indicating that reduced LPC effects were associated with higher trust levels. These implications of these results are discussed in the context of the P3b and P600 ERP components. Additionally, there were no effects of model transparency on ERP results or subjective trust ratings, suggesting that trust is primarily developed through direct observation of model performance. Our results contribute to understanding the neural mechanisms underlying trust in automation, highlighting the potential of ERP methodologies to advance our understanding in this domain.

## Significance statement

This research sheds light on the complex phenomenon of how users develop trust in artificial intelligence (AI) and other automated decision aid systems. Right now, there is widespread inconsistency in how trust is measured across different studies, leading to confusion in the literature about how systems can be designed so that users develop appropriate trust in them. It is important to understand how people learn to trust the output of automated decision aids because they are becoming increasingly prevalent in almost all areas of modern life, and both over-reliance and under-reliance on automated aids could have disastrous consequences. By investigating how explanations of model behavior influence user trust, the study reveals that simply providing textual information about a model’s biases does not necessarily help users better understand its performance. Instead, people tend to rely more on their direct experiences with the model. This finding is crucial as it challenges the common belief that increased transparency in AI outputs will automatically lead to increased appropriate trust. Understanding these dynamics is essential for developing more effective and reliable AI systems that people can trust, especially in high-consequence decision-making situations relevant to national security, like threat detection in overhead imagery or baggage screening, as well as other critical areas of life, like anomaly detection in medical diagnostics. By improving how we understand and measure the development of trust in AI, we can foster better decision-making and ultimately ensure safer and more effective use of this emerging technology.

## Introduction

The use of automated decision-aid systems to help human decision-makers is growing rapidly. The goal of such systems is to enable the end user to make better and faster decisions than they otherwise would make without the system’s suggestions. Many of these systems incorporate machine learning (ML), artificial intelligence (AI) or other types of complex algorithms whose inner workings may be difficult or impossible for users to understand. However, it is critical that users develop an understanding of the circumstances under which the system is trustworthy and should be relied on, and the situations in which it is not. In other words, effective human-system performance relies on the users having *appropriate* trust in the system. In order to determine whether or not users have developed appropriate trust, researchers must first define quantifiable metrics that capture the multi-faceted concept of human trust in an automated system. Currently, the measurement of trust in AI is inconsistent across the literature, and few methods have considered the use of brain electrophysiological signals (c.f. Ajenaghughrure et al. 2021; De Visser et al., 2018). In the present study, we integrate brain and behavioral measures to test whether the human electroencephalogram (EEG) can be leveraged to identify a brain-based measurement of trust in automation. Unlike overt behavioral measures that ask users to rate their subjective trust, which interrupt natural task performance and make the user self-aware of their trust levels, electrophysiological measures can be collected unobtrusively. Using this approach, the multi-dimensional, continuous neural signals of interest are automatically elicited, while the user processes information from the AI system.

In this introduction, we will first give a brief overview of current theoretical models of trust in automation models, introduce the use of electrophysiology to measure trust in automation, and finally set up the current experiment.

### How is trust defined?

Most automated decision-aid systems operate without transparency into their underlying workings, meaning that users do not usually have information about how these black-box systems make recommendations or why a given recommendation has been made. As such, it is critical to understand how human users develop appropriate trust in automated decision-aid systems, especially in high-consequence domains. Unfortunately, *defining* and *measuring* human trust in automation are non-trivial tasks. There are two widely cited models of trust that researchers typically look to when setting out to define trust in automation. One of these, Mayer et al. (1995)’s process model of trust, defines trust as a multi-faceted latent concept that includes factors such as the perceived trustworthiness of the model, the user’s propensity to trust, the perceived risk of the situation, and the level of risk-taking in the relationship. The second of these, Lee and See (2004)’s model of trust, focuses on the dynamic nature of trust in automation and emphasizes how factors such as context, automation characteristics, and individual cognitive processes impact trust over time. Although these models share many similarities, they ultimately define trust differently. As noted by Wisniewski et al. (2024), Mayer et al. (1995) define trust as an *intention* (e.g., a willingness of the user to be vulnerable to the output of the system), whereas Lee and See (2004) define trust as an *attitude* (e.g., the attitude that using the system will provide a benefit to the user in an uncertain situation). These different starting definitions of trust make it difficult to compare outcomes when studies use one definition or the other. Moreover, Jacovi et al. (2021) differentiate between *warranted* trust versus *unwarranted* trust, or trust that is either derived from model trustworthiness or not, as another way to determine the appropriateness of user trust. Wisniewski et al. (2024) instead propose a simplified model of trust that synthesizes across these multiple definitions. They posit that *perceived risk* of the situation and *perceived trustworthiness* of the automation come together to produce a *trust decision,* which is measurable by the user’s actions. Under this formulation, the actual risk and trustworthiness can be compared to the perceived risk and trustworthiness to determine *appropriate* and *inappropriate* trust and distrust. In light of this model, it is exceedingly important to measure the perceived risk of the situation and perceived trustworthiness of the system from the user’s point of view, in addition to the actual risk and trustworthiness, in order to evaluate trust appropriateness.

After the user makes an initial evaluation of whether they trust a model’s suggestion or not, there is typically an additional step of whether, and to what extent, to incorporate that suggestion into their own decision-making process (e.g., Love et al., 2024; Steyvers et al., 2022). There is a wide range of factors that may influence how much a person relies on a model suggestion when making their final decision. These factors could be situational, like the risks associated with an incorrect choice, or idiosyncratic, for example, the user’s confidence in their ability to perform the task, predisposition to trust automation, or individual experience with a particular model. Lai and colleagues (2023) provide an excellent review of the state of the literature regarding AI-assisted human decision-making. In general, their review summarizes that the state of this literature is quite fragmented, due to the use of a variety of different decision tasks, metrics of human performance, datasets, and AI elements across studies. Given that our current study does not include a decision-making element (participants observed a model’s performance without having to take action on it), a further review of this literature is beyond the scope of this paper.

### What makes a model trustworthy?

Given a focus on a user’s perception of the trustworthiness of a model, we must consider the factors that influence this perception. Jacovi et al. (2021) formalized a theory of trust that hinges on two key points for the formation of human trust in AI: (1) The human user is vulnerable to the AI’s decisions (e.g., there is risk involved to the user if they rely on the AI), and (2) the human user can anticipate the AI’s decisions. This theory leads to the prediction that it is not *model accuracy* per se that builds trust, but rather the user’s ability *to anticipate* model performance under certain circumstances. The user’s expectation of model performance could be as simple as knowing that the model will achieve a particular accuracy for one input type (e.g., pictures of dogs) but not another (e.g., pictures of cars). The authors suggest that knowing these details regarding patterns of model performance will help users more accurately anticipate the model’s response for specific inputs, which will in turn increase appropriate trust.

This theoretical framework sets up several testable predictions, which will form the basis of the current study. The first prediction is that, given the same model performance, exposing people to an explanation of the model’s behavior will help them more accurately anticipate the model’s performance on individual stimuli. This prediction stems from the assumption that users can integrate a text explanation of the model’s performance into their real-time prediction of how the model will perform. We will test this assumption in the current experiment. Furthermore, Jacovi et al.’s (2021) framework suggests that there may be a difference in how people assess model performance if they implicitly learned about that performance via observing the model’s outputs for different types of stimuli versus being explicitly given explanations of its performance. We will also test this prediction in the current experiment.

### How is trust measured?

There are many approaches to measuring trust, including self-report, behavioral, and psychophysiological measures (see Kohn et al., 2021 for a comprehensive review). The majority of studies on trust in automation or AI rely on self-report measures. Popular self-report measures include the Trust of Automated Systems Test (TOAST; Wojton et al., 2020) and the Propensity to Trust scale (Merritt, 2011), among others. Many self-report survey tools conflate different definitions of trust. For example, they may ask questions about both trust as an attitude, as in Lee and See’s (2004) definition, and trust as an intention, as in Mayer et al.’s (1995) definition. Trust is often defined behaviorally by measuring whether the user complied with or relied on the automated assistant in their decision-making process. For example, Love et al. (2024) measured how much people adjusted their initial response in a perceptual judgment task toward an AI-generated suggestion. Interpreting behavioral reliance as trust could be problematic, however, because the same binary *reliance* decision could be associated with different underlying psychological states or attitudes related to trust, such as high perceived trustworthiness in one individual and low perceived trustworthiness in another individual. These two situations would be indistinguishable based on behavioral responses alone. As such, researchers have been moving toward incorporating psychophysiological techniques (e.g., eye-tracking, EEG) into the measurement of trust, with the goal of identifying continuous, noninvasive metrics of trust that can be measured in real time and provide a more nuanced view into the underlying cognitive processing that supports trust.

Ideally, researchers would incorporate multiple measures to find converging evidence, and this is indeed a popular approach. For example, Tenhundfeld et al. (2024) had participants interact with an automated self-driving golf cart and recorded self-report, behavioral, and physiological measurements (in this case, heart rate [HR] and heart rate variability [HRV]) of trust in the automated agent. However, they found little convergence across measures and suggested that the different timescales of these measurements posed a challenge to their integration. On the other hand, De Visser et al. (2023) did find convergence between multiple measures of trust in an automated parking task. Specifically, users spent more time monitoring a parking system, as evidenced by eye movement recordings, that they reported as trusting less than one they trusted more. There are at least two possible explanations for these differences in findings. First, the eye movements to the automated parking system in De Visser et al. (2023) were measured in real time, whereas HR and HRV are measured at longer timescales (20 s post-incident). Secondly, there is a clear theoretical connection between eye movement behaviors and attention: More time spent viewing the system indicated that the person was paying more attention to it (Rayner, 2009). Heart rate and HRV are more generalized measures and may be more reflective of stress or workload than trust alone (Charles & Nixon, 2019; Järvelin-Pasanen et al., 2018; Kim et al., 2018), so it may not be surprising that they did not track with other measures of trust. Regardless, even these two examples demonstrate the lack of clarity in the field as to which trust measures are most appropriate to use under which circumstances, and importantly, how they relate to the underlying cognitive processes they represent.

### Using event-related potentials to measure trust

In the current study, we will use the event-related potential of the EEG signal to measure trust in an automated agent’s responses. EEG provides a continuous, multi-dimensional measure of brain electrical activity recorded at the scalp during task performance. Event-related potentials (ERPs) are segments of the EEG time-locked to stimuli of interest. The timing, polarity, and amplitude of ERPs elicited by certain types of stimuli inform researchers about the cognitive processes elicited by those stimuli in different tasks. ERPs could make an ideal tool to study trust in automation because they are elicited obligatorily when participants process a stimulus, which means that their collection does not require that participants stop their task to overtly answer questions. One challenge with using ERPs to measure trust is the need to time-lock to a stimulus event of interest. This can prove difficult in operationally relevant environments wherein users interact with a system for an extended period of time. A second challenge with using ERPs is the functional specificity of the ERP components relative to the task. In other words, because there is not a “trust in automation” ERP component, we will examine how other aspects of stimulus processing are modulated with manipulations of model trustworthiness, in an attempt to connect how trust in a model impacts the way people process its outputs, especially its errors.

One family of ERP components, the error-related negativity (ERN) and error positivity (Pe), has previously been used as a proxy measure of human trust in automation (De Visser et al., 2018). The ERN is elicited when a person makes a response error in a forced-choice response task (Falkenstein et al., 1991; Gehring et al., 1993). This component is frontally distributed and peaks around ~ 50 ms (ms) after the response is made to the error. The ERN is typically followed by a positive deflection, known as the Pe, which is also observed over frontal channels and peaks ~ 200–300 ms post-error response. The ERN and the Pe are believed to come from different neural sources and represent different processing mechanisms (Falkenstein et al., 2000). Several theories exist as to the functional specificity of the ERN (Gehring et al., 2012; Gehring et al., 2018), though the most common theories suggest that it reflects error processing, conflict monitoring, and/or cognitive control. The ERN may be linked to behavioral adaptation following an error, though evidence for this theory is mixed (see LoTemplio et al., 2023, for review). The significance of the Pe is less clear. Falkenstein (2004) proposed that the Pe may be related to affective processing of the error, error awareness, or post-error slowing. A review by Overbeek et al. (2005) found evidence across studies that the amplitude of the Pe (but not the ERN) tended to track with the degree of awareness of the error or the salience of the error but found little consistent evidence for the affective processing or post-error slowing hypotheses of the Pe. Relationships between the ERN and Pe and other response-monitoring components have also been proposed (Ullsperger et al., 2014).

Despite the ongoing debate regarding the processing mechanisms that these the ERN and Pe reflect, they are consistently observed in speeded forced-choice tasks. One such task commonly used to elicit the ERN and Pe—and the task employed in the current experiment—is the flanker task (Eriksen & Eriksen, 1974). In this task, a person makes a speeded button-press response to a centrally presented stimulus. One stimulus–response combination is mapped to one hand, and the other stimulus–response combination is mapped to the other hand (e.g., S = right hand, H = left hand). The “flanking” stimuli presented on either side of the target are either response-congruent (e.g., SSSSS) or response-incongruent (e.g., HHSHH). The typical pattern of results is that responses are faster and more accurate when the flanking stimuli are response-congruent versus response-incongruent. In this case, the response-incongruent flanking stimuli activate the incorrect response, which slows response times to correct responses and/or causes participants to make errors. When people make errors in the flanker task, they elicit an ERN and Pe, time-locked to the error response (Falkenstein et al., 1991; Gehring et al., 1993).

Extensions of this paradigm have shown that people also elicit an ERN and Pe when they *watch* someone else commit an error in the Flanker task (De Bruijn & von Rhein, 2012; van Schie et al., 2004). In a related study, Carp et al. (2009) manipulated the perceived similarity between an observer and confederate “committing” the errors in the flanker task. They found a reduced ERN, but increased Pe amplitude, when the observer perceived themselves as being more similar to the confederate, suggesting a difference in sensitivity to errors committed by similar others. Somon et al. (2019) also found error-related ERPs as participants observed what they believed to be a human or automated system perform a modified flanker task. The amplitude of some of these components was reduced for system- versus human-made errors, again suggesting differences in sensitivity to system versus human errors. Relatedly, Kang et al., (2025) found reduced feedback effects in a hyperscanning EEG study (e.g., recording EEG from two participants simultaneously watching or performing the same task) when participants observed their friends complete a gambling task, relative to watching a stranger complete the same task. The authors interpreted these attenuated effects in friend dyads as showing reduced performance monitoring when in a trusting social situation with a friend versus a stranger.

Notably, the ERN has also been elicited when participants observed a “machine learning algorithm” make mistakes in a modified flanker task (De Visser et al., 2018). Because the current study leverages De Visser et al. (2018), their study design and findings will be described in detail. De Visser et al. (2018) had participants perform a flanker task, intermixed with blocks in which they observed a simulated machine learning algorithm “perform” the flanker task. They manipulated block-wise model reliability (60% or 90% reliability) and model credibility (i.e., participants read a story that the model was either created by experts or novices, even though models presented across these two conditions were identical). The credibility manipulation was intended to influence the trustworthiness of the model, with the prediction that people should trust the model made by experts more than the model made by novices, even if in reality, the performance was matched across expert and novice conditions. Participant-generated ERN and Pe amplitudes from the performance of a flanker task were compared to those elicited by observed model-generated errors (called oERN and oPe, for *observational* ERN and Pe). They predicted that ERNs would be observed to both participant- and model-generated errors. Moreover, the authors predicted that oERNs and oPes would be biggest to errors made by the expert model in the 90% reliability condition (because they were the least expected), smallest to errors made by the novice model in the 60% reliable condition (because they were the most expected), and somewhere in the middle for the other two conditions (expert-made 60% reliable and novice-made 90% reliable models). However, their results showed that oERN amplitude tracked only with trial accuracy: It was bigger (more negative) for error than correct trials and did not differ across the 60% and 90% reliability conditions. The oPe showed effects of both trial accuracy and model reliability: It was larger (more positive) for error versus correct trials, and also for 90% versus 60% reliable models. There was no effect of the model credibility manipulation on ERPs. This is likely because the actual model performance was the same across these two blocks, despite what was told to participants. Participants also completed a trust in automation question after each observation block. Interestingly, results showed that the amplitude of the oPe elicited by model errors was correlated with self-reported trust, such that higher trust ratings were associated with larger amplitude oPe. The authors interpreted this to mean that errors made by the model were more salient and/or attentionally orienting when the person had higher trust in the model, hence resulting in larger amplitude oPe components.

The lack of a credibility effect in De Visser et al. (2018)’s results was likely driven by the fact that the two credibility conditions were identical performance-wise. Regardless of whether participants were told that they were viewing an expert- or novice-made model, the two models performed with the same accuracy. As such, ERPs to model errors only reflected the model’s performance, not the participants’ belief about whether it was created by an expert or novice. In other words, the cover story alone was not enough to overcome participants’ implicit learning based on their experience of actual model performance.

In the current study, we will also include a transparency manipulation, using a broad definition of transparency suggested by Varshney (2022) which encompasses model performance information. In this manipulation, some participants will be told about an aspect of model bias but others will not. We want to test whether participants can use information about model performance, given to them before they interact with the model, to modulate their expectations about how it will perform in real time. Importantly, what differentiates our study from De Visser et al. (2018) is that the bias that participants are told about will actually be present in the model’s performance. Recall that in De Visser et al. (2018), the credibility manipulation was in name only: The actual model performance of these two conditions was matched. In our study, the model will indeed be biased to perform better for some stimuli in the biased blocks. This will allow us to test whether participants can integrate information about model bias in a top-down manner to influence their moment-to-moment evaluation of model performance for those stimuli. This manipulation also enables us to test Jacovi et al. (2021)’s prediction that providing users with more information about the model will help them better predict its performance. If so, we would expect to see a difference in ERPs elicited between the groups who learned the information about model bias and those who did not. If we do not observe differences between these conditions, it will provide further evidence that the user’s experience with the model may override transparency information in their moment-to-moment interpretation of model output.

### Current experiment

The goal of the current experiment was to use ERPs to measure a person’s trust in an automated agent’s task performance. Using a flanker task, we collected ERPs time-locked to the participant’s response in order to measure the error-related negativity (ERN) and post-error positivity (Pe) associated with participant-made errors. This was included to help participants become acclimated with the task. We also used a modified flanker task to collect ERPs time-locked to model-generated errors, as in De Visser et al. (2018). Building on the framework proposed by Jacovi et al. (2021), we tested the hypothesis that both model *reliability* and model *predictability* contribute to trust, and that providing people with *explanations* of model bias before exposure to model performance will help them better calibrate their trust. Specifically:

1. We tested the impact of model reliability by including two levels of block-wise model reliability (60% and 90%) to test the difference between oERN and oPe amplitude elicited by models with different levels of reliability. We predict that errors made in the 90% reliability block will elicit larger oERN and oPe amplitude than errors in the 60% reliability block because they are less expected.
2. We manipulated the predictability of model output by having the model perform more accurately with one target letter than the other in some blocks (biased blocks) but not others (balanced blocks). This manipulation is included to mimic instances in the real world in which a model may perform better for some types of stimuli than others, which users need to notice to appropriately calibrate trust. We predict that for the biased blocks, errors made to the “better letter” will elicit larger oPe amplitude than errors to the “worse letter” because errors to the “better letter” are less expected.
3. We manipulated the provision of explanations by incorporating a between-subjects transparency manipulation: Half of the participants were informed of the model bias, including which letter it performs better for, and half were not. We predict that if users could incorporate model explanations in a top-down way, then errors made by the model in the biased blocks, especially those to the “worse letter,” should be more expected to people in the informed block and thus should elicit smaller oERNs and oPes.

Trust will be measured in two ways in the current study: first, by inferring trust from ERP effects elicited across conditions, and second, by overtly measuring subjective trust through self-report measures. This application of the error-monitoring paradigm provides an opportunity to apply psychophysiological techniques to the study of trust in automation. From an experimental perspective, it allows us a way to observe human evaluation of model-generated errors unobtrusively and provides clear time-locking points (which are typically not available during more naturalistic interactions with an automated assistant). From a broader theoretical perspective, understanding how people initially process and evaluate model errors could provide important insights into how users develop appropriate trust by allowing us to infer which performance monitoring and attentional processes are brought online when people encounter model errors across conditions. It is important in an operational setting that users can quickly identify and overcome automation errors. We know that people are vulnerable to complying with model-made errors in a variety of other experimental settings (Matzen et al., 2024; Stites et al., 2023; Stites et al., 2021). Because many other factors may come into play between the initial evaluation of a model-made error and the decision to ultimately comply with that model or not, we have chosen in the current experiment to focus on the first wave of processing when an error is committed. By utilizing a well-studied experimental paradigm in the flanker task, we can clearly establish links between the trust literature and the existing cognitive psychophysiological literature.

## Method

### Participants

Twenty-four people participated in this experiment. All participants were employees of Sandia National Laboratories and were compensated for their time at their usual hourly rate. Of the participants, 11 were female and 13 were male. Their average age was 40 years (range = 24–82).

### Materials

For the performance flanker task, stimuli consisted of five-letter arrays, with the target letter (S or H) in the center and two flanker letters on each side. On congruent trials, the flanking letters matched the target letter (SSSSS or HHHHH). On incongruent trials, the flanker letters did not match the target letter (HHSHH or SSHSS). The target letter indicated the appropriate response hand, which was counterbalanced across participants. All letters were white on a black background. Note that although this task was based on the task used by De Visser et al. (2018), the stimuli were different. While De Visser et al. (2018) used arrows (< or >) in their flanker task stimuli, we chose to use letters to minimize the chances of the participants misunderstanding the information about the model bias. We believed that instructions such as “The model is more accurate for S than for H” would be easier to understand and remember than “The model is more accurate for < than for >.”

For the observation flanker task, the stimuli consisted of a single white target stimulus shown in the middle of the screen, followed by the model’s response shown in yellow. On model-correct trials, the model’s response matched the target stimulus (S–S or H–H). On model-incorrect trials, the model’s response did not match the target stimulus (S–H or H–S). On review trials, participants saw an asterisk (*) in the middle of the screen following the reporting of the model’s response, indicating that they were to respond as to whether the ML model had been correct on the previous trial or not.

Model reliability was manipulated at the block level by adjusting how frequently the model provided the correct response (e.g., 60% accurate, 90% accurate). Model bias was also manipulated at the block level by having the model perform more accurately for one target letter than the other (in the biased blocks) or having the model perform equally for both target letters. Importantly, the overall model accuracy was held constant in the biased blocks. For example, in the “Balanced 60%” block, the model was 60% accurate for both S and H targets. However, in the “Biased 60%” block, the model was 80% accurate for S targets, 40% accurate for H targets (or vice versa), with overall 60% accuracy. Sample stimulus counts for each block shown in Table 1. The letter for which the model was more accurate was counterbalanced across participants. Each person completed four observation blocks across this combination of factors, the order of which was counterbalanced across participants using a Latin squares design.

**Table 1:** Stimulus counts for model correct and incorrect decisions in the observation blocks. This table assumes that “S” was the “better letter” in the biased blocks. Note that “H” was the “better letter” in the biased blocks for half of participants

Model bias	Model reliability (%)	Model correct (S Target)	Model correct (H Target)	Model incorrect (S Target)	Model incorrect (H Target)	Review trials	Total (excluding Review)
Balanced	60	48	48	32	32	16	160
Biased	60	64	32	16	48	16	160
Balanced	90	72	72	8	8	16	160
Biased	90	76	68	4	12	16	160

Subjective trust ratings were collected at the end of each block by asking participants a series of three questions: “To what extent do you trust the algorithm’s ability to identify [S/H/ALL] targets, on a scale from 1 [very low trust] to 5 [very high trust]?”.

### Procedure

After obtaining informed consent, each experimental session started with providing participants with the following instructions: *In this experiment, sometimes you will perform a task and other times you will watch a machine learning algorithm perform that same task. A machine learning algorithm is a computer program that can be trained to perform tasks by showing it data. You will observe a total of 4 machine learning algorithms perform the task. We are testing these algorithms for future research, and your goal is to help us decide which ones we should trust most in the future… In some of the blocks, you will watch a machine learning algorithm perform the task. The algorithm was presented with a string of S's and H's similar to what you will see, but you will only be shown the center letter from that string. The center letter that the algorithm was presented with will be shown in WHITE. The algorithm's response will be shown in YELLOW.*

The instructions were followed by practice blocks for both the performance and observation flanker tasks. Participants had to achieve at least 70% accuracy for the performance block to move forward; otherwise, they were required to repeat the block. After the practice, each participant completed six blocks of work in the following order: one performance block, two observation blocks, one performance block, and two observation blocks. The order in which the type of observation blocks appeared was counterbalanced across participants.

**Fig. 1:**
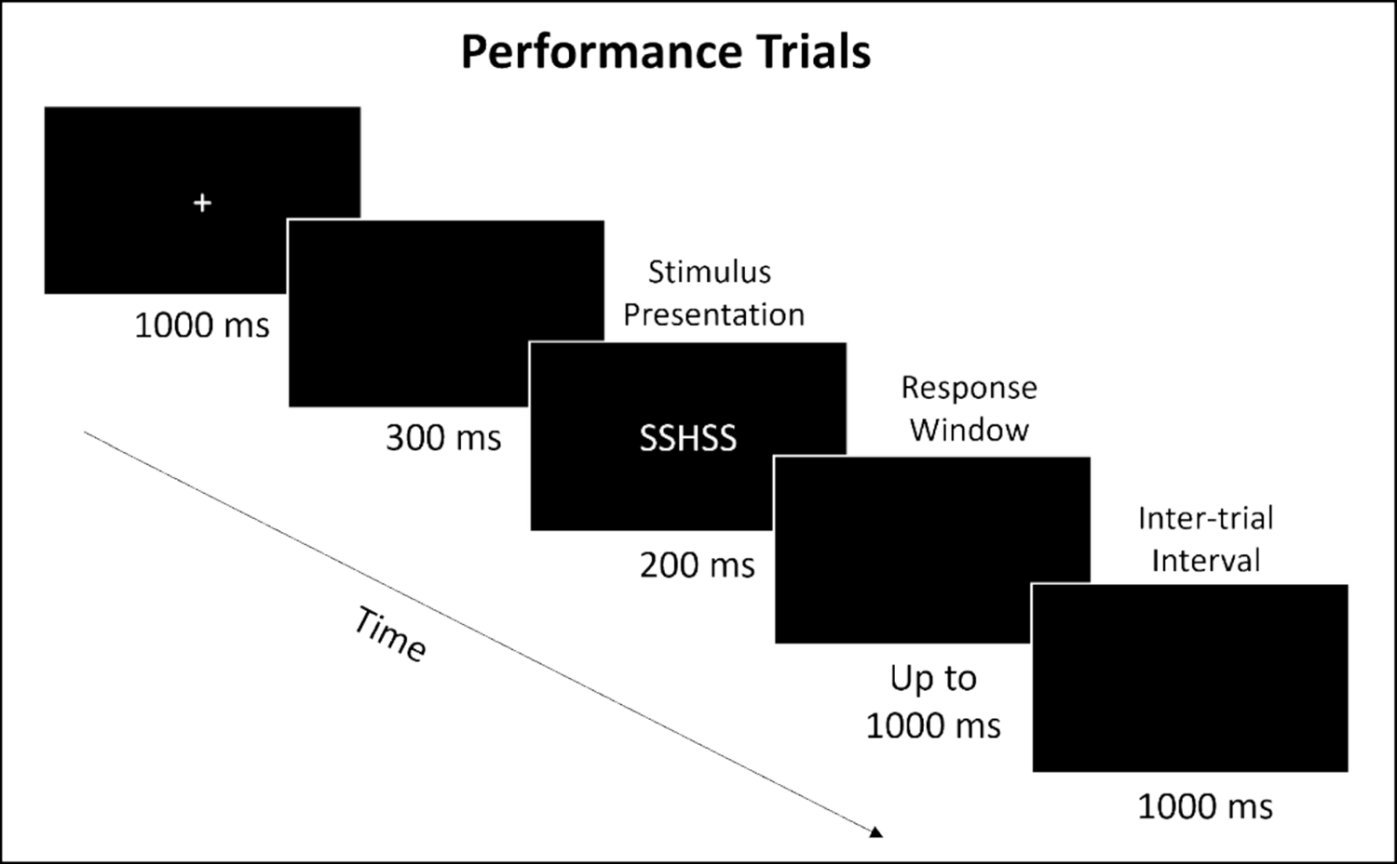
Stimulus timing for performance flanker trials

In the performance block (see Fig. 1), each trial began with a fixation cross that was presented for 1000 ms, followed by a 300 ms blank screen, and then, the stimulus was presented for 200 ms with a 1000 ms response window. The task was to respond as quickly and accurately as possible to the central target letter using the button box. The response hand-mapping to target letters was counterbalanced across participants. After the response was recorded, there was a 1000 ms inter-trial interval before the fixation cross indicating the start of the next trial appeared. Participants completed 160 trials in the performance block in a random order: 80 congruent and 80 incongruent, each with 40 S targets and 40 H targets.

**Fig. 2:**
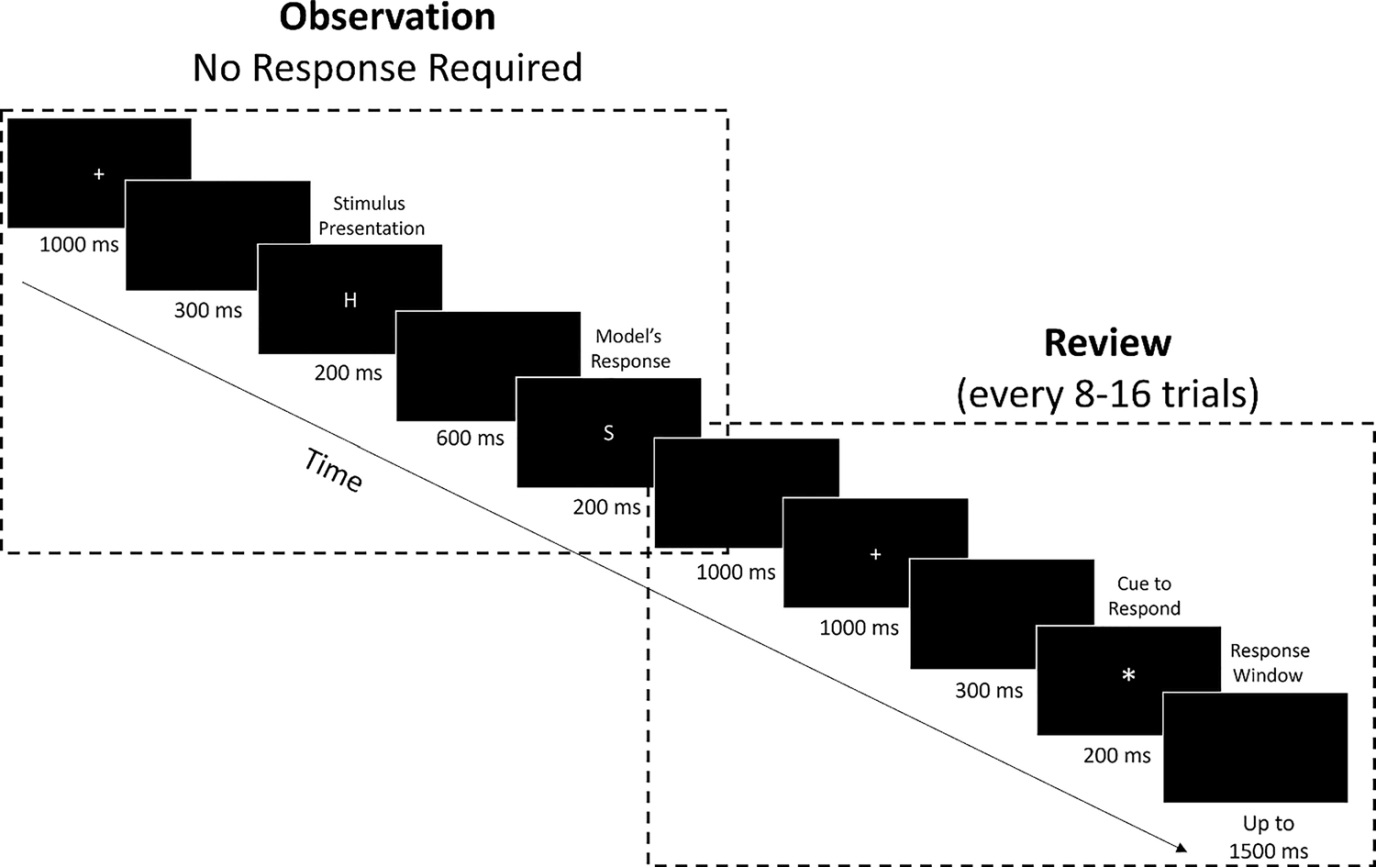
Stimulus timing for trials in the observation blocks

Before each observation block, participants received the instructions: *In the next block, you will WATCH a machine learning algorithm perform the flanker task. This is model number (#).* Participants in the “model information condition” received the additional instruction: *This algorithm models “H” better than “S.”* The structure of observation trials is shown in Fig. 2. Each trial began with a fixation cross that was presented for 1000 ms, followed by a 300 ms blank screen. Next, the target stimulus was presented for 200 ms, followed by a 600 ms blank screen before the presentation of the model’s response, which was shown for 200 ms. There was a 1000 ms inter-trial interval before the start of the next trial. Participants were not required to respond to these trials. For review trials, the target stimulus was replaced by an asterisk “*” for 200 ms, followed by a 1500 ms response window. Participants responded by pressing the “up” button on the button box if the model was correct on the previous trial and by pressing the “down” button if the model was incorrect on the previous trial. There were 16 review trials, which appeared every 8–16 trials. Participants were given a self-timed break between blocks. Each experimental session took no more than 180 min.

### EEG recording and analysis

The ongoing encephalogram (EEG) was recorded from 32 active silver/silver-chloride electrodes using the ANT Neuro waveguard cap arranged in the 10/20 layout. Electrodes were referenced online to a ground electrode near Cz and were re-referenced offline to the average of the left and right mastoids. A bipolar eye channel was created by placing electrodes on the left infraorbital ridge and above the left eye, referenced to each other, to monitor for blinks. A second bipolar eye channel was created by placing electrodes on the outer canthus of each eye, referenced to each other, to monitor for horizontal eye movements. Impedances for scalp electrodes were kept below 25kΩ. The continuous EEG was recorded to hard disk at a sampling rate of 250Hz.

All data processing was completed using the EEGLAB (Delorme & Makeig, 2004) and ERPLAB (Lopez-Calderon & Luck, 2014) toolboxes for MATLAB. For the performance trials, epochs of EEG data were time-locked to the response and were taken from 100 ms pre-response to 900 ms post-response. For the observation trials, epochs of EEG data were time-locked to the onset of the ML response stimulus and were taken from 100 ms pre-stimulus onset to 900 ms post-stimulus. ICA blink correction was applied to the data; first, segments of the EEG more than two seconds away from any stimulus were removed; next, ICA weights were calculated for each of the scalp channels; the topographic weights of these maps were visually inspected, and the components corresponding to eye blink artifacts were selected for each person individually and removed from the data. Next, epochs containing artifacts from signal drift, eye movements, eye blinks, muscle activity, or other types of noise were rejected offline before averaging, using thresholds selected for each participant through visual inspection of the data. Trial loss averaged 21.5% (range 0–76.7%). Two individuals were removed from analyses due to low trial numbers. Artifact-free ERPs were averaged by stimulus type after subtraction of the 100 ms pre-stimulus baseline. Prior to statistical analyses, ERPs were digitally filtered with a low-pass filter of 30Hz.

Based on De Visser et al. (2018), ERN and Pe values were measured from the Fz electrode. Time windows were selected based on viewing the grand average waveforms and selecting the time window that best captured each effect, collapsing across participants and conditions. For the performance trials, ERNs were calculated as the mean amplitude at Fz from 50 to 100 ms post-response. The Pe was calculated as the mean amplitude at Fz from 200 to 250 ms post-response. There was also a large, late posterior positivity observed following errors in all conditions. Although this was not reported by De Visser et al. (2018), the effect was large enough that it warranted inclusion in our analysis. We will refer to it as the Late Positive Component, or LPC. The LPC was calculated at the mean amplitude at Pz from 250 to 350 ms post-response. For the observation trials, the oERN was calculated as the mean amplitude at Fz from 125 to 175 ms post-stimulus onset, the oPe was calculated as the mean amplitude at Fz from 200 to 250 ms post-stimulus onset, and the LPC was calculated as the mean amplitude at Pz from 500 to 700 ms post-stimulus onset.

## Results

### Behavioral performance

#### Flanker task

Participant accuracy on the flanker trials was higher for congruent than incongruent trials (see Table 2). Accuracy data were analyzed using a binomial linear mixed-effects model, with the fixed effect of flanker congruence (congruent, incongruent) and random intercepts for subjects, using the lme4 R package (Bates et al., 2015). Results showed a significant effect of flanker congruence (*ß * = − 0.91, z = − 6.25, *p* =  <.001), with higher accuracy for congruent than incongruent trials. Response times on flanker task performance also showed an effect of congruence, with slower RTs for incongruent than congruent trials (also shown in Table 2). Results from a one-way within-subjects ANOVA showed a significant main effect of flanker congruence on RTs (*F*(1, 23) = 156.9, *p* <.001). These results replicate previous flanker task performance, with more errors and slower responses when flankers were incongruent than congruent.

**Table 2:** Accuracy and response times (RT) for performance trials

Measure	Flanker congruence	Mean	SD
Accuracy	Congruent	0.98	0.02
	Incongruent	0.96	0.03
RT (ms)	Congruent	485	86
	Incongruent	531	92

### Review trials

Review trials were interspersed throughout the model performance blocks to ensure that participants were attending to the model’s responses. When indicated with an asterisk, participants responded as to whether the model was correct on the previous trial or not. Accuracy for these trials was high (see Table 3). Data were analyzed using a binomial linear mixed-effects model, with the fixed effects of model reliability (60%, 90%), model bias (biased, balanced), and their interaction, as well as random intercepts for subjects. The significance of these effects was assessed by calculating the analysis of deviance using the Anova() command from the car package (Fox & Weisberg, 2019). This analysis found that the main effects of model reliability and model bias were not significant (bias: *Chi-sq* =.82, *p* =.36; reliability: *Chi-sq* =.0007, *p* =.97), but there was a significant interaction (*Chi-sq* = 7.83, *p* <.01). Pairwise comparisons were calculated using the emmeans package (Lenth, 2023), using the Tukey correction for the familywise error rate. These pairwise comparisons showed that accuracy was significantly higher in the biased 60% condition than the balanced 60% condition (z = 2.66, *p* <.05); no other comparisons reached significance. The high performance on the review trials suggests that participants were actively attending to the model’s responses in the observation blocks. Differences observed across these blocks in the ERPs would be unlikely to be driven by reduced attention.

**Table 3:** Mean accuracy on review trials in the model observation blocks

Reliability	Bias	Mean	SD
60% Reliability	Balanced	0.93	0.14
	Biased	0.97	0.06
90% Reliability	Balanced	0.96	0.12
	Biased	0.94	0.14

### ERP results

#### Performance Flanker ERPs

**Fig. 3:**
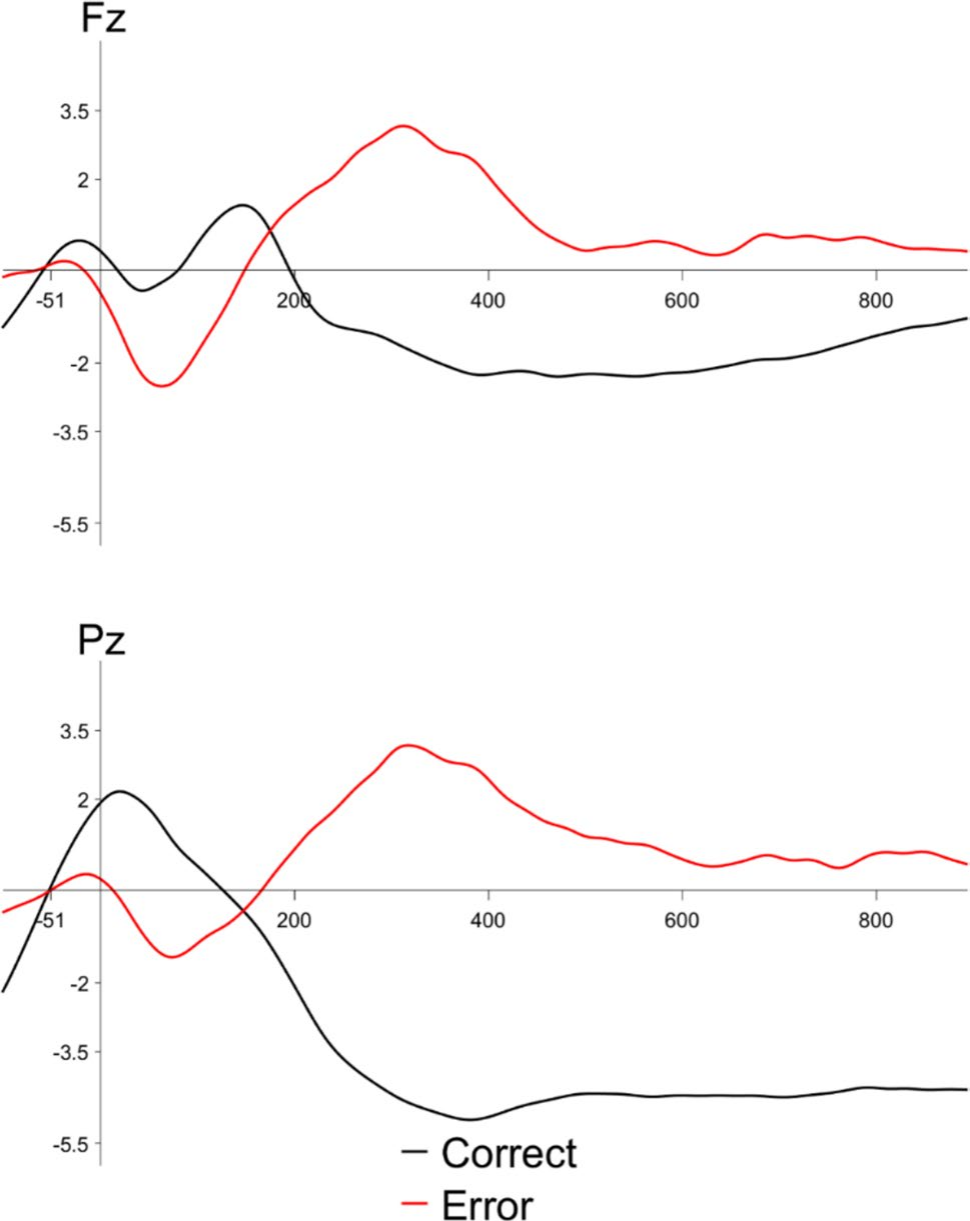
Grand average performance ERPs time-locked to response onset to all correct trials (black lines) and errors (red lines) at electrode Fz (top) and Pz (bottom)

We observed an ERN to error versus correct responses in the flanker performance block, shown in Fig. 3. A paired-samples *t* test revealed a significant effect of accuracy (*t*(23) = 2.65, *p* =.01), reflecting a larger amplitude ERN to error than correct trials. We also observed a Pe to error versus correct responses in the performance block. A paired-samples *t* test revealed a significant effect of accuracy (*t*(23) = −3.81, *p* <.01). Additionally, we observed an LPC over posterior channels to errors made in the flanker task relative to correct trials. A paired-samples *t* test showed a significant effect of accuracy (*t*(23) = −7.32, *p* <.01).

### Model observation ERPs

#### Model Reliability × Model Errors

**Fig. 4:**
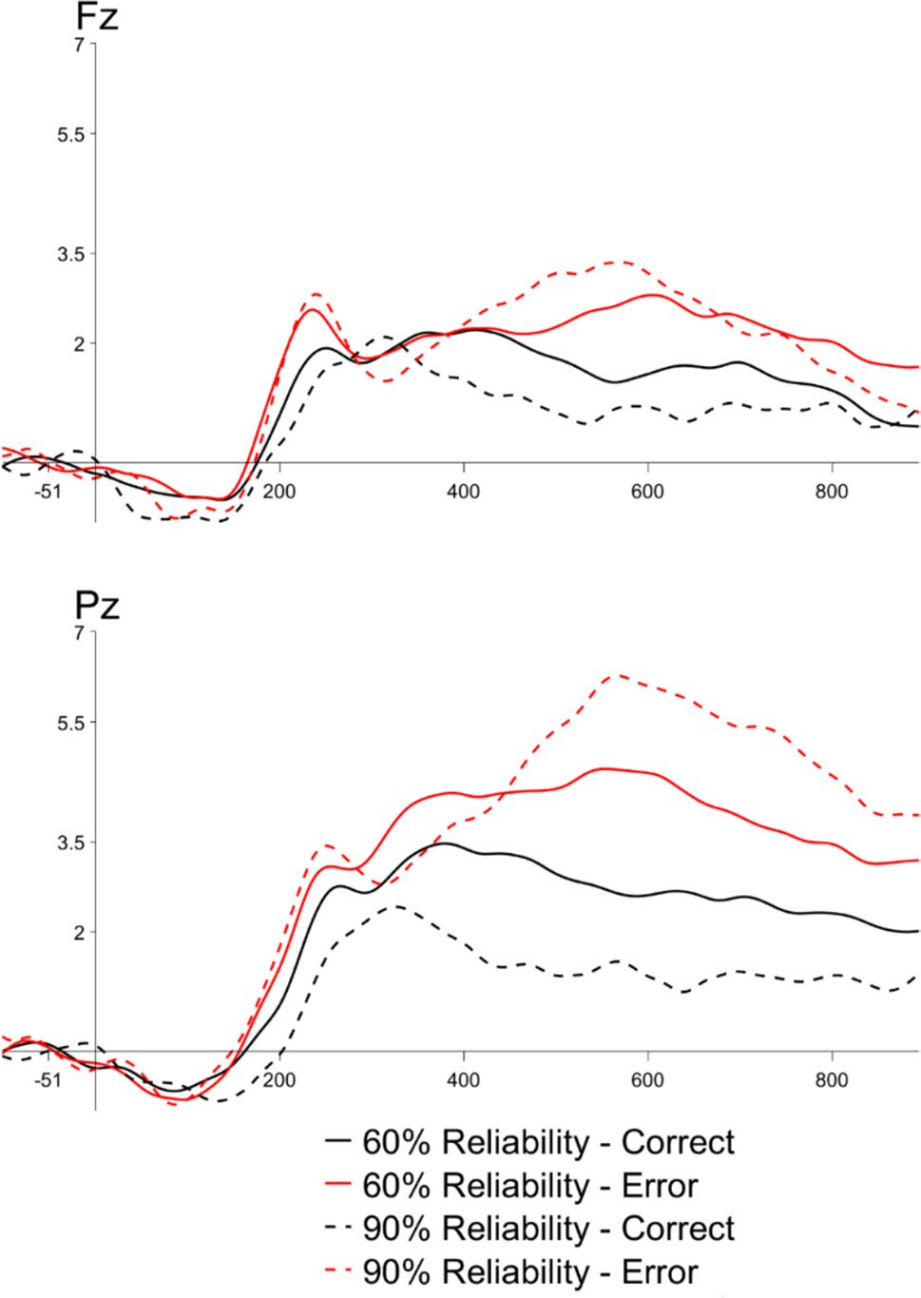
Grand average observation ERPs to model errors (red) vs. model-correct (black) trials, split by model accuracy (60% = solid, 90% = dashed), at Fz (left) and Pz (right) electrodes

Next, we examined ERPs elicited by model errors and correct trials across the two levels of block-wise model reliability (see Fig. 4). We conducted a 2 × 2 within-subjects ANOVA with the factors of model reliability (2: 60%, 90%) and trial-level accuracy (2: correct, error) separately for the ERN, Pe, and LPC time windows. For the ERN, we again did not observe effects of model reliability (*F*(1,23) = 0.81, *p* =.38), accuracy (*F*(1,23) = 0.25, *p* =.62), nor an interaction (*F*(1,23) = 0.02, *p* =.90). We did find a significant Pe to errors made by the model, *F*(1,23) = 20.42, *p* <.001), with no effects of model reliability (*F*(1,23) = 0.32, *p* =.57) nor interaction (*F*(1,23) = 2.37, *p* =.14). For the LPC, we found a significant effect of trial accuracy (*F*(1,23) = 21.06, *p* <.001), as well as a significant interaction between trial accuracy and model reliability (*F*(1,23) = 8.98, *p* <.01). Follow-up pairwise t tests showed that the LPC was larger (more positive) for error than correct trials in the 90% reliability condition (*t* = −5.48, *p* <.001), but error and correct trials did not significantly differ in the 60% reliability condition (*t* = −2.14, *p* =.16).

#### Letter Type × Model Errors in Biased Blocks

**Fig. 5:**
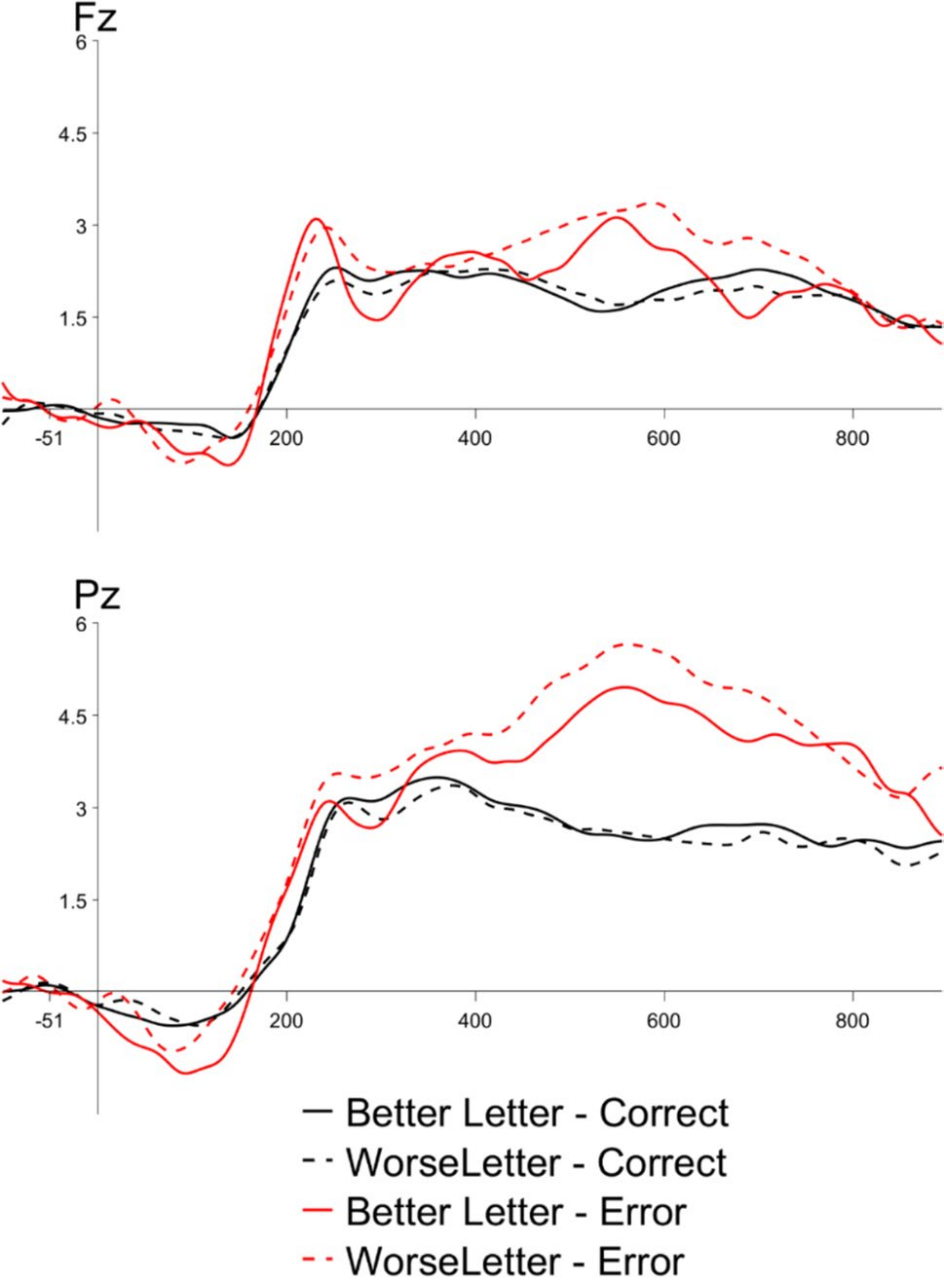
Grand average observation ERPs to the better letter (solid) and worse letter (dotted) in the biased model conditions, collapsing across 60% and 90% reliability, for Fz (left) and Pz (right)

Next, we examined the impact of model bias and individual letter performance on ERPs. In our study design, there were two target types: S and H. In the balanced conditions, the simulated ML model performed with equal reliability for the S and H targets. In the biased conditions, the simulated ML model performed with a higher level of reliability for one letter versus the other, while the block-level reliability was held constant. For this analysis, we will report on data from a combined dataset, collapsing across the biased 60% and 90% reliability conditions. (Note: the results are the same whether we consider 60% and 90% separately, or together.) ERPs are shown in Fig. 5. Two-way ANOVAs were conducted with the within-subjects factors of letter type (2: better letter, worse letter) and trial accuracy (2: correct, error), separately for the ERN, Pe, and LPC. For the ERN, there were no significant effects (all *F* < 2.26, all *p* >.15). For the Pe, there was a significant effect of accuracy (*F*(1,23) = 17.90, *p* <.001), with a larger amplitude LPC for model errors relative to correct trials, with no effects of letter type or interaction (all *F* < 1, all *p* >.35). Similarly, for the LPC, there was a significant effect of accuracy (*F*(1,23) = 20.04, *p* <.001), with a larger amplitude LPC for model errors relative to correct trials, with no effects of letter type or interaction (all *F* < 1, all *p* >.35).

#### Effect of Transparency Information on ERPs in Biased Blocks

**Fig. 6:**
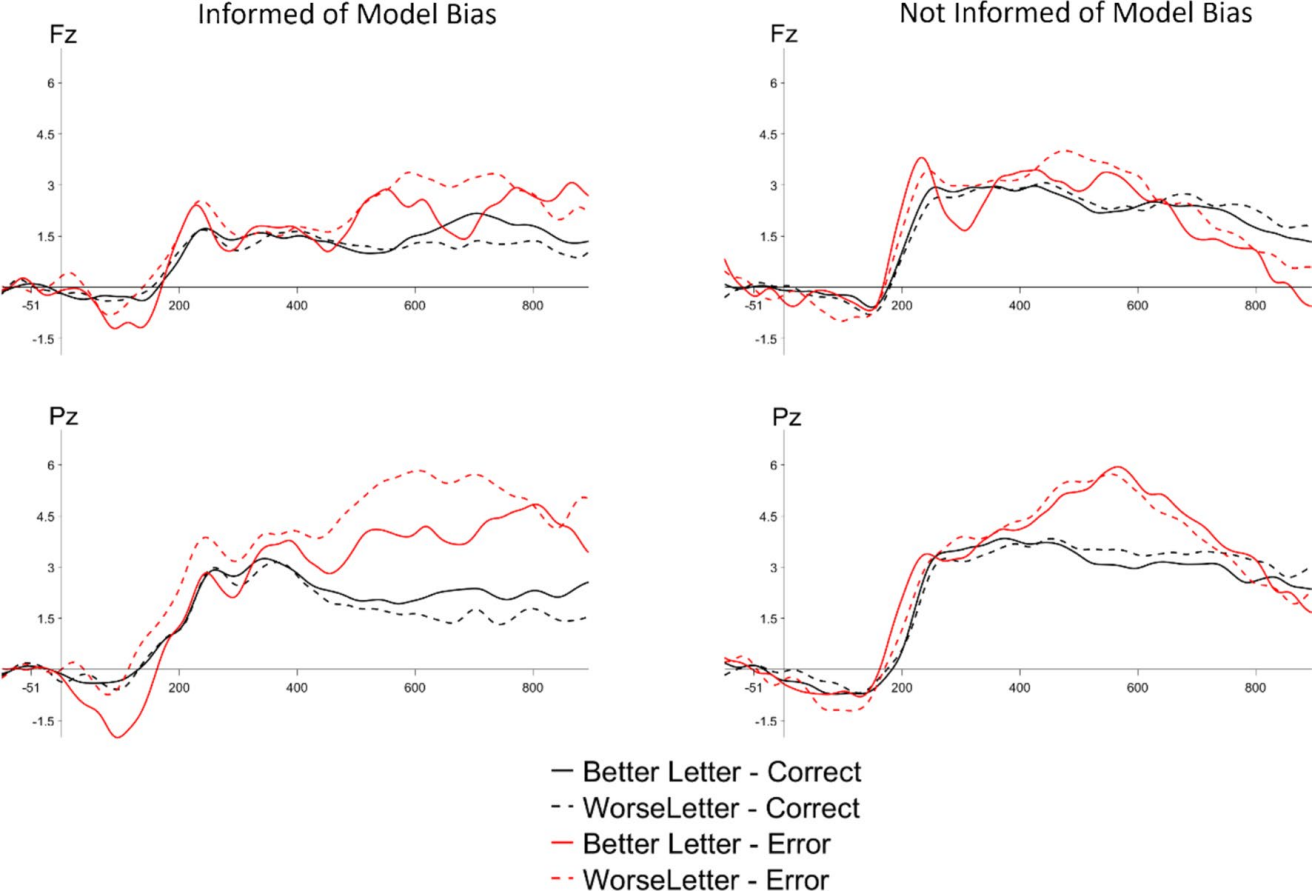
Grand average observation ERPs to model errors made to the better versus worse letters in the biased conditions only, plotted separately for participants informed of model bias versus those not informed of model bias

Next, we tested the effect of our transparency manipulation. In our design, half of the participants were informed that the model was more reliable with one letter than the other in the biased blocks. These participants were also told *which* letter the model would respond more reliably to. We tested to see whether any differences were observed to ERPs elicited by model errors across these groups, which would help answer our question of whether people could use transparency information in a top-down manner to modulate error assessment in real time (see Fig. 6). The 60% and 90% biased conditions were again collapsed for the purpose of these analyses. ERP amplitudes were analyzed with a $2 \times 2 \times 2 \times$ mixed-effects ANOVA, with the within-subjects factors of letter type (2: better, worse) and trial accuracy (2: correct, error), and the between-subjects factor of informed condition (2: informed, not informed). Models were run separately for the ERN, Pe, and LPC. Results showed, for both the Pe and LPC, main effects of trial accuracy (Pe: *F*(1,22) = 17.64, *p* <.001; LPC: *F*(1,22) = 19.85, *p* <.001)) with no other effects or interactions (all *F* < 1.28, all *p* >.28). Both groups of participants elicited a Pe and LPC to model errors, regardless of which letter the error was made to, or whether they were informed of the model’s bias or not. No effects were observed for the ERN.

### Subjective trust ratings

#### Overall

**Fig. 7:**
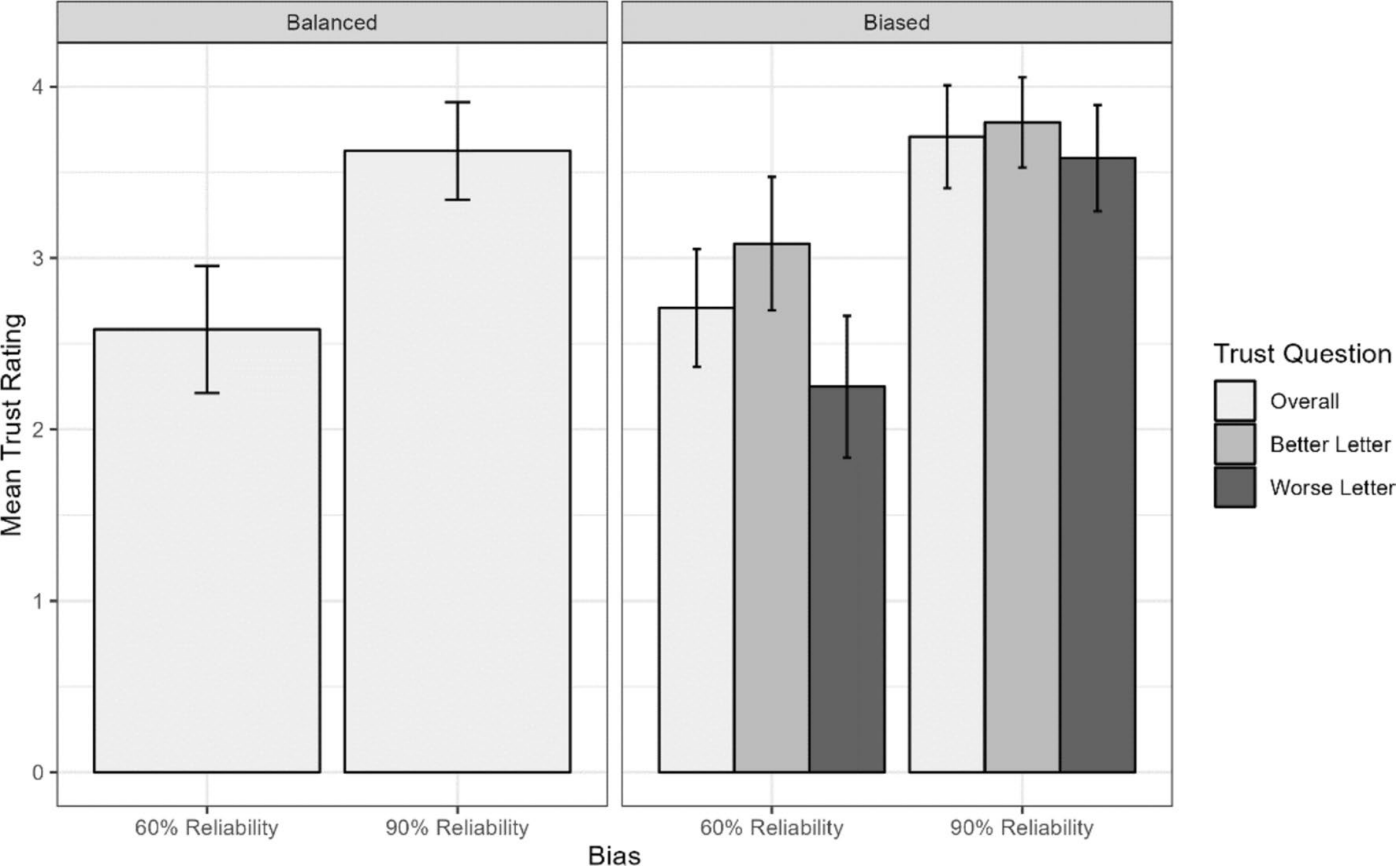
Subjective trust ratings for each observation block. Error bars represent 95% confidence intervals

Participants rated their subjective trust in the model after each observation block. Mean values are shown in Fig. 7. Participants expressed higher subjective trust ratings in the 90% reliability condition than the 60% reliability condition. This was confirmed by separate analyses conducted on the ratings from the balanced and biased blocks. A one-way within-subjects ANOVA on overall ratings from the balanced conditions showed a main effect of model reliability (*F*(1,23) = 38.4, *p* <.001). A two-way within-subjects ANOVA on ratings from the biased conditions, with factors of model reliability (2: 60% vs. 90%) and question type (3: better letter, worse letter, overall rating), showed main effects of both model reliability (*F*(1,23) = 37.01, *p* <.01), question type (*F*(2,46) = 7.01, *p* <.01), as well as a significant interaction (*F*(2,43) = 6.01, *p* <.01). Follow-up pairwise comparisons within each model reliability condition indicated that the better letter was rated as significantly more trustworthy than the worse letter in the 60% reliability condition (*t* = 5.02, *p* <.0001). There were no differences between letters in the 90% accurate condition.

#### Effects of transparency information on subjective trust ratings

**Fig. 8:**
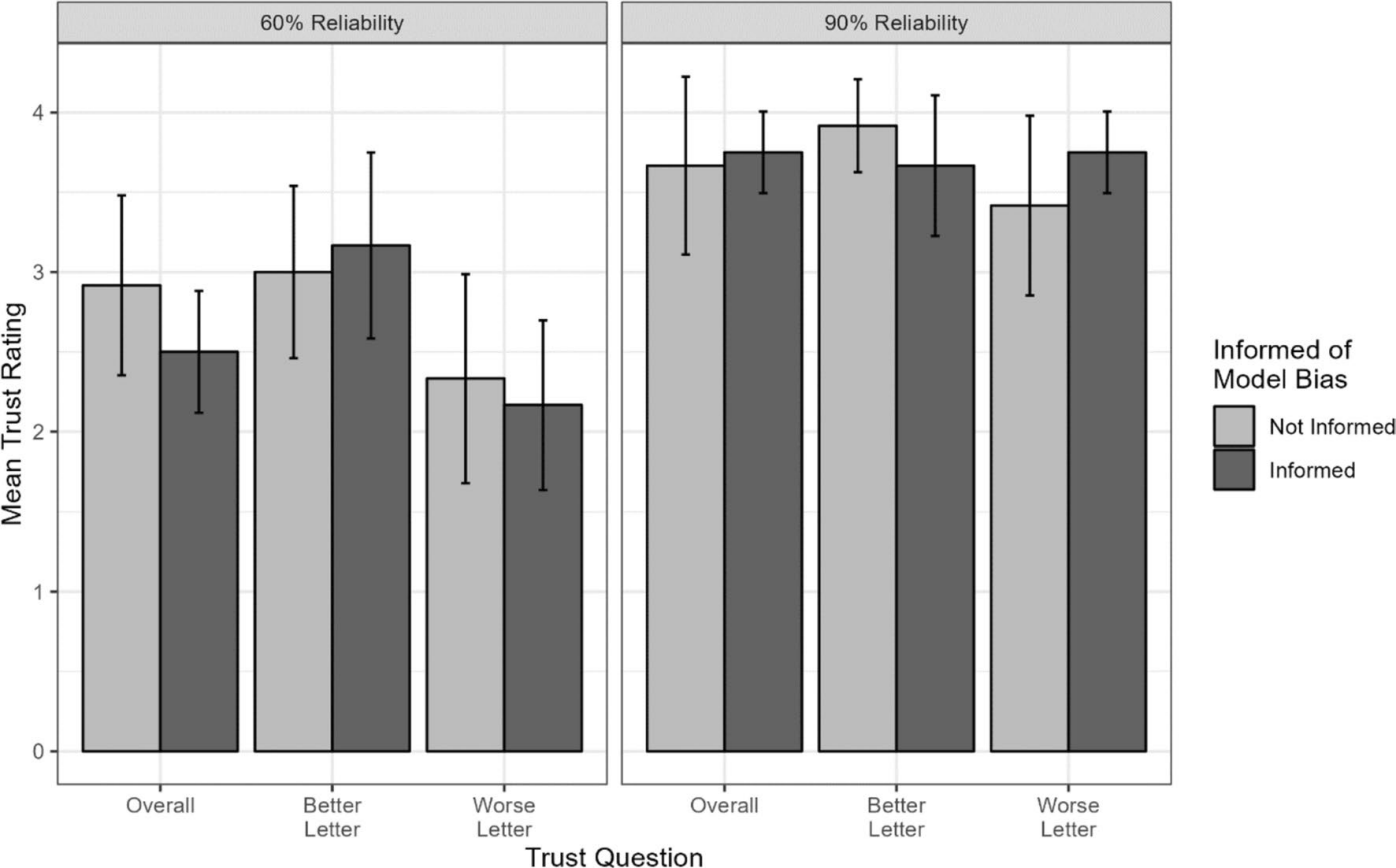
Mean trust ratings for biased conditions, split by which letter the rating was given for and whether participants were informed of the bias manipulation or not. Error bars represent 95% confidence intervals

Next, we tested whether being told before starting the block that the model would perform better for one letter than the other impacted the trust ratings participants later provided for the model’s performance with that letter. We tested trust ratings from the 60% biased and 90% biased blocks separately. For the 60% biased block, a 3 × 2 mixed ANOVA with the within-subjects factor of question type (3: better letter, worse letter, overall) and the between-subjects factor of informed status (2: informed, naïve) showed a main effect of question type (*F*(2,44) = 11.54, *p* <.01), with no main effect of informed status nor interaction (all *F* < 1.41, all *p* >.25). Pairwise comparisons between question types showed that both the better letter and overall ratings were higher than the rating for the worse letter (better vs. worse: *t* = 4.80, *p* <.01; overall vs. worse: *t* = 2.64, *p* =.03). For the 90% biased block, an identical analysis showed no significant effects (all *F* < 1.82, all *p* >.18). These results indicate that informing participants of model bias before their interactions with the model did not ultimately impact their trust in the model after exposure to the model’s performance. Results are shown in Fig. 8.

### Correlations between trust and ERP effects

Recall that De Visser et al. (2018) found that people’s trust in the model was correlated with the amplitude of the oPe to errors. Specifically, they found that larger oPe amplitude to model errors was associated with higher trust, suggesting that model errors were more attentionally orienting for people with higher trust in the model. In our attempt to replicate these findings, we examined the correlation between ERP effects elicited by model errors in each block (e.g., Balanced 60% Reliable, Balanced 90% Reliable, etc.) and trust ratings given by the participant at the end of that block. ERP effects were calculated by subtracting mean ERP amplitude elicited by correct responses from the mean ERP amplitude elicited by errors (error–correct) for each participant in each condition. Correlations were calculated separately for the ERN, Pe, and LPC components. Results are shown in Table 4. We found a significant negative correlation between LPC effects and trust scores in the Biased 60% Reliable condition (*r* = −.486, *p* =.032); no other effects were significant.

**Table 4:** Correlations between mean ERP effects and trust score in each block. Bonferroni corrections were applied to account for multiple comparisons within each component

	Reliability (%)	Bias	*R*	*t*	*p*
ERN	60	Balanced	0.055	0.258	1
		Biased	0.124	0.588	1
	90	Balanced	− 0.240	− 1.160	0.518
		Biased	− 0.219	−1.050	0.609
PE	60	Balanced	0.082	0.383	1
		Biased	− 0.309	− 1.520	0.284
	90	Balanced	− 0.126	− 0.595	1
		Biased	− 0.267	− 1.300	0.416
LPC	60	Balanced	− 0.070	− 0.329	1
		Biased	− **0.486**	− **2.610**	**0.032**
	90	Balanced	− 0.012	− 0.057	1
		Biased	0.016	0.077	1

## Discussion

The goal of this study was to use ERP measurements collected while participants watched a simulated ML algorithm perform a modified flanker task to test the sensitivity of brain-based measures of trust in automation to the factors of ML reliability, bias, and explanation of model behavior to users.

Behavioral results showed high performance on both the performance and observation flanker tasks. In the performance flanker task, we observed the typical pattern of results: higher accuracy and shorter response times for congruent relative to incongruent trials. In the observation flanker task, participants responded to review trials with 93–97% accuracy. This result shows that that they were closely attending to the stimuli in this task and able to correctly report whether the model had made an error or not on the preceding trial. Based on this high behavioral accuracy on the review trials, we feel confident that any differences observed in the ERPs elicited across observation blocks were not driven by attentional differences in monitoring.

Turning to the electrophysiological outcomes, we observed the canonical ERN and Pe to errors relative to correct trials on the performance flanker task. This was in line with our predictions. We also observed a significant posteriorly distributed late positive component (LPC) to errors relative to correct trials. The elicitation of such an LPC is typical in flanker tasks (e.g., Hajcak et al., 2005; Holroyd et al., 2004; Scheffers & Coles, 2000) even though the ERN/Pe complex tends to be the main focus of analyses in these papers. Previous work has interpreted this late positive component to likely be a P300, which is a positive-going deflection of the ERP observed over posterior channels, peaking around 300–400 ms post-stimulus onset and elicited by infrequent targets (originally reported by Sutton et al., 1965). The P300, including its eliciting conditions and functional significance, will be discussed in greater detail below.

Next, we examined ERPs on observation trials, in which participants observed a “machine learning model” perform the flanker task. We tested predictions made by Jacovi et al.’s (2021) paper that making model errors more predictable, and thus less surprising, would increase trust in the model. Based on findings from De Visser et al. (2018), we predicted that we would see an oERN and an oPe to errors made by the model. Of particular interest was how the brain would respond to more or less predictable model errors. Because there is not an ERP component that specifically reflects trust in automation, we instead evaluated known cognitive processes elicited by model errors to ask questions like: *Does the person notice when the model made an error? How were those errors evaluated? Did those processes change when they had more information about likely errors?* We predicted that the oERN and oPe components would be larger to less expected errors, specifically to errors in the 90% versus 60% block and to errors made to the “better” versus “worse” letters. We also predicted that if participants were able to integrate the model accuracy information they received before the block to better anticipate model errors to certain stimuli (as predicted by Jacovi et al. (2021)), then their ERPs to more common errors in the biased block should be smaller, because those errors would be more predictable.

Overall, we did not find differences in oERN amplitudes to model-made errors across any condition. The lack of an oERN effect for error trials was inconsistent with our predictions and also with De Visser et al. (2018)’s findings. Recall that in De Visser et al. (2018), oERNs were observed to all model errors relative to correct trials—leading to the question of why our results did not also show this difference. We posit several possible explanations. First, there are some fundamental differences between the two studies with respect to the stimuli. In De Visser et al. (2018), participants saw a centrally presented arrow (< or >), followed by an asterisk on one side of the screen to indicate which “hand” the ML algorithm had “responded” with. In contrast, our participants saw a centrally presented target letter in white followed by a centrally presented response letter in yellow. As discussed above, we chose to use letters rather than arrows to make it easier to convey information about the model performance to the participants. Although the tasks were very similar otherwise, it is possible that the letter stimuli elicited different types of processing than the arrows used by De Visser et al. (2018). However, we did not anticipate the change in the stimuli to lead to changes in the ERP responses, so it is unclear whether and how this difference might have impacted our results.

As a second explanation for the lack of differences on the oERN, previous work has shown that in flanker tasks, a “correct response negativity” can also be observed alongside the ERN, especially when participants are unsure of their response accuracy (e.g., Scheffers & Coles, 2000; Coles et al., 2001; Pailing & Segalowitz, 2004). For example, Scheffers and Coles (2000) observed negativities in the ERN time window for “unsure” *correct* responses relative to “sure” correct responses. This and similar findings have been interpreted as showing that uncertainty in the correctness of the response may give rise to a CRN. It is possible that participants may have maintained some uncertainty regarding the correctness of model responses, thus eliciting a CRN on correct trials in addition to an ERN on incorrect trials. Variability in this effect could obscure differences across conditions. Other work has also shown variability in the ERN response in response-monitoring paradigms that closely mirrored our own. For example, Somon et al. (2019) used a response-monitoring paradigm in which participants monitored the performance of a “human” and “automated” agent perform a flanker task. They did not find differences in the oERN (which they called an N200) component between error and correct trials in what they deemed their “easy” condition, in which participants saw only the central stimulus of the flanker task, as in our design. They *did*, however, observe differences on this negativity in their “hard” condition, in which participants saw both the target and the flanking stimuli. Moreover, Carp et al. (2009) found a reduced oERN effect as the perceived similarity between the participant and confederate increased, suggesting that additional factors beyond simple error detection impact that amplitude of the oERN. Finally, Kang et al., (2025) found reduced frontally distributed feedback effects when participants observed a friend perform a gambling task, relative to watching a stranger perform the task, suggesting reduced monitoring in higher trust social situations. Clearly, more work is needed to understand the experimental conditions that influence the elicitation and size of the oERN, and potentially CRN, in response-monitoring tasks. We posit that this will be especially useful in the study of trust in automation, as more human–machine interactions take on a supervisory role, with the human monitoring the automation for errors.

We consistently found a significant oPe to model errors relative to correct trials, although the oPe was insensitive to the model reliability or bias conditions. In previous work, the oPe has been shown to differentiate between different levels of error awareness and is larger for errors that the participant explicitly notices versus those they did not (Nieuwenhuis et al., 2001; Shalgi et al., 2009). It is interesting that the size of the oPe effect did not change with error probability across the 60% and 90% reliability blocks, was unaffected by the transparency manipulation, and was not correlated with subjective trust measures. Our findings seem to support the idea that, at least in this response-monitoring context, the oPe reflected conscious awareness of the model’s error (Overbeek et al., 2005).

We also observed a large oLPC to model-made errors relative to correct trials. Unlike the oPe, the amplitude of the oLPC was sensitive to model reliability: oLPCs were larger to errors in the 90% reliability condition (i.e., when errors were less frequent) than in the 60% reliability condition (i.e., when errors were more common). This effect was not reported by De Visser et al. (2018), so we did not initially predict it. However, this pattern of results is similar to other late positive components frequently observed in the ERP literature, such as the P300. One paradigm frequently used to elicit a subtype of the P300, the P3b, is the oddball task. In this task, participants monitor a steam of stimuli for rare “oddball” targets and make a categorical decision or button-press to these oddballs (Sutton et al., 1982). The amplitude of the P3b is inversely related to subjective probability of targets, with larger positivities elicited by targets that are less probable or expected (Polich, 2012). The amplitude of the P3b has been shown to be larger for targets that are salient, being attended to, or task-relevant (Johnson & Donchin, 1980), as well as in response to targets that are easily classified relative to those for which categorization is less clear (e.g., Polich, 1987). One of the predominant theories of the P3b is that it represents context updating (Donchin, 1981). The idea of context updating is that as people interact with their environment, they build a mental model of that context. When new information comes in that differs from the ongoing context, it triggers a revision of that model and these revision processes elicit the P3b.

Another well-studied late positive component is the P600, which was originally observed to syntactic violations in language comprehension (Osterhout & Holcomb, 1992), and shares a similar posterior scalp distribution to the P3b but with slightly later peak latencies (around 600 ms, though again this timing can vary). It has been found that the P600 is not only elicited by syntactic violations, but also grammatical structures that are more difficult to process (Gouvea et al., 2010), as well as non-linguistic stimuli, such as phrase structure violations in music (Patel et al., 1998). Multiple theories have been proposed as to the functional specificity of the P600 (Kuperberg, 2007; Brouwer et al., 2012), suggesting that it may reflect revision or repair processes that are initiated when the processing system notices an error in combinatorial processing. Recently, Sassenhagen et al., (2020) provided evidence suggesting that the P600 is likely a variant of the P3b. In general, both components are elicited by less probable stimuli that may trigger the need to re-evaluate or update the ongoing context.

Interpreting our oLPC findings in light of the P3b and P600 studies, our findings align in that the oLPC observed in our study was larger for the less frequent errors (e.g., those made by the 90% reliable model) than for the more frequent errors (e.g., those made by the 60% reliable model). These results seem to indicate that in our study, the brain treated model errors more like salient rare targets (that were somewhat surprising, and thus less predictable) and less like errors per se (in that they did not elicit an ERN). Contrary to Jacovi et al.’s (2021) prediction, trying to make the errors more predictable by telling participants to expect them in the biased blocks did not make them less surprising to the brain, at least at the level measured by the LPC/P300. More broadly, there is an interesting theoretical connection between trust and surprise with respect to interpretation of model outputs. If a model’s output was always perfectly predictable to the user, it would be hard to justify its existence. (Does a model add value if it completely replicates a user’s responses?). It seems that in order to have a situation in which trust is needed, the model’s outputs should be surprising to the user at least some of the time. In our study, all of the surprising outputs were model errors. It could be interesting in future studies to explore cases in a more complex decision-making scenario in which a model gives an unexpected but correct answer (similar to a reader receiving an unpredictable but grammatical ending to a highly constraining sentence, e.g., Federmeier et al., 2007), to see how people interpret these “surprising but right” outputs. Moreover, it will be critical to understand how those initial “surprise” signals do or do not get translated into eventual reliance on the model. Future work is needed to further disentangle how predictability of model outputs contributes to model trust across AI-assisted decision-making contexts.

Additionally, we found a negative correlation between the size of the oLPC effect in the biased 60% reliability block and trust ratings for this block. In other words, people who elicited smaller oLPC effects to errors during this block later rated themselves as having higher trust in the model’s performance for that block. We posit that the stimulus reevaluation or context-updating processes that elicited the oLPC were not triggered as strongly in these people, and so observing model errors was not as detrimental to their development of trust in the model. It could be that they devoted fewer attentional resources to attending to these errors and updating their mental model to degrade their trusting state. It could also be the case that model errors were interpreted as being less salient, leading to higher trust ratings in these participants. Additionally, we hypothesize that this effect was not present in the 90% reliability conditions because trust was overall much higher in these blocks, limiting the variability in the response. Based on the model’s high reliability, it may have been obvious that people should trust it, leading to a weaker relationship between ERP responses and trust ratings in this block.

From a theoretical perspective, then, our findings replicate De Visser et al. (2018) in that: (1) we found evidence that people did differentiate model-made error versus correct trials, as evidenced by trial accuracy effects on the oPe component instead of the oERN, and (2) we found evidence that people were sensitive to *likelihood* of model errors, as evidenced by model reliability effects on the oLPC instead of the oPe. The differences between the stimuli used in the two experiments (arrows versus letters) may have altered the processing mechanisms that people engaged while evaluating the model responses, thus changing the ERP components on which we observed effects. Nevertheless, our findings converge to suggest that the use of human electrophysiology may provide a fruitful avenue for the study of trust in automation.

### Impacts of model transparency

According to Jacovi et al. (2021), it is not model accuracy per se that builds trust, but the user’s ability to anticipate model performance under certain circumstances. We tested this prediction by manipulating whether people were informed of model bias to ask the following question: Given the same model performance, will exposing people to an explanation of the model’s behavior *before* they interact with it (e.g., model is more accurate for X stimulus type than Y) help them make more accurate real-time predictions about the model’s performance on individual stimuli? We did not find any group-wise differences between the informed relative to naïve conditions, neither in the ERPs elicited to errors by these groups nor in the subjective trust ratings they provided to these conditions. As such, our results do **not** support the prediction that providing people with model performance information (e.g., model will perform better for X versus Y stimuli) in advance of interacting with the model helped them better tune their trust of the model or anticipate outcomes for specific stimuli (e.g., did not attenuate error-related ERPs to more predictable errors). Our findings instead suggest that users cannot integrate a text explanation of the model’s expected stimulus-level performance into their real-time interpretation of model outputs. Regardless of whether they were informed of the model’s bias or not, participants elicited the same ERP responses to model errors made to both the better and worse letters. Interestingly, the descriptions of expected model performance also did not impact subjective trust ratings. Explicitly telling participants that the model performed better for *S* versus *H* did not result in those participants rating the model as more trustworthy for *S* than *H* responses—even though the model performed as described, so they received both explicit and implicit cues to model reliability.

These results are consistent with De Visser et al. (2018) in showing that people develop their expectations about the model’s performance through observation rather than from text-based descriptions. In De Visser et al. (2018), it did not matter whether people were told that the model was made by experts or novices; after they interacted with the model for a single block, their trust measures tracked only with model performance. In our study, it did not matter that in some blocks, the model made twice as many errors for one letter type than the other, and that we explicitly told people as such—both their ERPs and subjective trust measures did not differentiate model performance on these two stimulus types. This lack of observed differences between conditions with and without explanations did not support the predictions made by Jacovi et al. (2021) that trust in a model can be explicitly manipulated by giving people more information about how it will perform.

More generally, our results suggest that text-based descriptions of model performance metrics provided asynchronously with model output may be difficult or impossible to integrate to help people develop appropriate trust. This is in line with prior work about the seeming disconnect between information provided to users up front and their ability to successfully use that information during interactions and decision-making with model output. For example, in Matzen et al. (2024), participants completed a visual search task with the aid of a simulated ML aid. The likelihood of different model error types from the ML aid was manipulated. Some participants were told that it was most important to correctly identify model misses, whereas others were told that it was most important to correctly identify model false alarms. Despite these explicit instructions, participants were unable to modulate their task performance to emphasize accuracy for one error type over another. Across a variety of studies, receiving additional information regarding model explanations or instructions for how to use model information rarely has had the desired effect of increasing appropriate trust and reliance. Rather, this literature has shown that: people do not or cannot use information about model bias to modulate their response patterns (Kunar & Watson, 2023; Matzen et al., 2024); increased transparency regarding model accuracy can produce inappropriate trust and/or over-reliance on a system (Kunar et al., 2024); explanations can improve model comprehension without impacting trust (Cheng et al., 2019); more detailed explanations do not always result in higher trust (Kizilcec, 2016); explanations of model confidence in data provenance can have mixed results on trust (Bruzzese et al., 2020); and people are more likely to comply with a model that provided them with more information, even if it is wrong (Stites et al., 2021, 2023).

Several recent reviews have tried to address the inconsistent effects of model transparency on human performance (e.g., Bhaskara et al., 2020; Rajabiyazdi & Jamieson, 2020; Van de Merwe et al., 2022). They note the complex relationships that exist between transparency, situation awareness, mental workload, and performance metrics within any given study. For example, increasing transparency may also increase mental workload (which is undesirable) but improve situation awareness (which is positive). Depending on the task the participant is performing, the way in which the transparency information is defined and displayed, and the specific metrics used to assess performance, the impacts of transparency may not always be straightforward. Indeed, in a meta-analysis of 89 experiments, Sargent et al., (2023) found positive impacts of transparency on metrics such as error rates, speed, trust, effective reliance, and situation awareness, but potentially negative effects in terms of over-reliance when the model is wrong. More work is needed to understand the interplay of these factors, especially in more complex and realistic task environments, and considering the impact of expertise (Bhaksara et al., 2020).

Together, these findings stand in contrast to many of the theories put forth by the of explainable AI (XAI) community, which posits that adding model features like explanations, transparency, or interpretability will increase human understanding of model outputs and therefore increase appropriate trust and reliance on such models (e.g., Angelov et al., 2021; Dwivedi et al., 2023; Xu et al., 2019). Many theories posit that providing users with specific types of model information will improve their understanding of the models and thus improve human decision-making performance. In order to generate hypotheses that can be tested in human subjects experiments, the field needs to come to a consensus regarding definitions for terms like *explainability* and *transparency*, so that human subjects experiments can implement these factors consistently across studies. Without agreed-upon definitions of these key concepts, inconsistencies in experimental results will continue to propagate. Our results add to the body of evidence reviewed above suggesting that the theories put forth by the XAI community may be disconnected from the cognitive needs of the end users. Specifically, transparency as defined in our study—giving users information about model performance for specific stimuli—did not impact any measures of trust, at least as measured in the initial stages of stimulus processing. One limitation of this study is that users did not have to make a decision using the model recommendations. This is quite different from more deliberative decision-making applications where AI-assistant suggestions may come into play. To study how people use AI suggestions in longer-term deliberations, an approach with EEG time–frequency analysis could be more appropriate (Fink & Benedek, 2014). With longer recordings over a more deliberative task, one could see (theoretically) how deliberations on more complex tasks are more or less “effortful” or attention-demanding with different types of AI explanations or transparency information. We argue that in order for the field of human–AI teaming to advance, practitioners in the XAI community need to work closely with cognitive psychologists and researchers in related fields in order to explicitly test how XAI impacts AI-assisted decision-making and the development of appropriate trust.

### Recommendations for using ERPs to study trust

Our findings imply that trust is developed and calibrated through user observation of model behavior. ERPs and other physiological tools are useful for studying how this process unfolds because they allow us to see evidence of users’ perception of the model accuracy and trustworthiness as that perception develops, without having to interrupt them to get self-report measures. More specifically, the oPe effect could be used to understand what types of model errors people notice or do not notice. This effect could be useful for researching how people develop their perception of model accuracy and whether or not that perception is correct. Using ERPs in this way would advance our understanding of trust in AI by providing a tool to measure perceived model accuracy over time, which likely plays a key role in determining perceived trustworthiness. It would also allow us to do more systematic research into what types of factors support the development of appropriate trust, for example, by uncovering factors that influence when people are aware of model errors, or when they should be more cautious about accepting its recommendations.

We saw a larger oLPC to model errors when participants were not expecting them, indicating that this effect could be used to study what happens with a model violates the user’s expectations. This is important because it is often difficult or impossible for users to articulate their expectations of model performance. If the oLPC observed in this study is indeed a variant of the P3b, then known properties of the P3b could be exploited to further our understanding of how user expectations are developed. Specifically, the P3b is sensitive not only to the *global* probability of a rare event (e.g., at the block level), but also the *local* probability (e.g., given the few preceding trials; Squires et al., 1976). This effect could be utilized to understand which aspects of the context are important for building user expectations of model performance at both the global and local levels. For example, we could use this to answer questions such as: What kind of model error is most salient to users, or what patterns of previous model performance (either immediate or broad) lead to the largest oLPCs to certain model outputs? Additionally, both the P3b and P600 are sensitive to effects of expertise. For example, P600 amplitude to overt violations is larger in people with higher language proficiency (Tanner et al., 2013), music proficiency (Granot & Donchin, 2002), and the P3b to mathematical equation violations differs with individual arithmetic skill (Dickson & Wicha, 2019). Given the difficulty of measuring expertise in many real-world tasks, variations in oLPC amplitude to the same model error types could be assessed across individuals as a tool to understand which aspects of expertise most strongly influence model error evaluation.

Finally, there was also a relationship between the oLPC effect and self-reported trust. Leveraging this link between subjective sensitivity to model accuracy and the oLPC effect could be a useful tool for validating self-report measures of trust (or developing new ones), so that researchers could use survey questions that link to a quantifiable psychophysiological mechanism. In other words, we do not always need to use ERPs to study trust in AI, but by using them we could potentially develop better behavioral and self-report measures of trust for use in future research. This would be better than the current scenario where researchers tend to use one-off surveys that are prone to bias because the researchers (and the participants) use muddled definitions of trust and/or interpret the meaning of the words “trust” and “trustworthiness” in different ways.

### Limitations and future directions

The flanker task used in the current experiment is an ideal starting place for exploring the development of a psychophysiological measure of trust in automation, given its simple design. That being said, it is quite different from the types of tasks analysts might do “in the wild” that would require them to rely on outputs from an automated assistant to make decisions. First, the stimuli were unambiguous as to whether the model was correct or not on each trial. It is possible that with more uncertain task with a less clear correct answer (e.g., visual target detection in noise), we may see more nuances emerge in how people evaluate information from the model or develop trust in it. Secondly, a significant limitation of our study was that participants did not have to make a decision with the model’s output in our task. This was intentional, as we were interested in studying the information evaluation phase of the decision-making process. The literature suggests that the measurement of the P3b ERP response reflects the initial stages of stimulus evaluation (Folstein & Van Petten, 2011) and does not reflect the full decision-making process (Fischer-Baum et al., 2014). As a result, we are unable to fully connect how predictability interacts with AI-assisted decision-making processes, particularly in controlled or top-down processing scenarios. It could be the case that trust decisions are more influenced by factors that come into play *after* information from a model is evaluated initially. There is a growing body of literature that seeks to understand how and to what extent humans consider the advice of a machine, or another human, when making decisions (e.g., Steyvers et al., 2022; Love et al., 2024; for review, see Lai et al., 2023). It will be interesting to learn which aspects of an AI system may influence someone to rely on a model output that they initially found surprising. More research is needed to understand the connection between initial information evaluation and the ultimate decision of whether to make a trust decision or not.

Third, our study did not include risk or vulnerability on the part of the participant. Several current theories of trust posit that some sort of vulnerability on the part of the user is necessary for the development of trust (e.g., Jacovi et al., 2021). Future studies could incorporate rewards or punishments based on model reliance decisions to further explore how the addition of risk impacts model information evaluation. Finally, this study only manipulated transparency insofar as participants were told about model bias characteristics at the start of the block. It is possible that other facets of transparency will prove to be more impactful to the development of trust, perhaps in different tasks or with different user populations. More work is needed to continue to work toward a fuller understanding of the relationship between model characteristics and trust development, and how these are reflected in psychophysiological signals.

## Conclusions

This study made progress toward identifying a psychophysiological marker of trust in automation. In a modified flanker task, we found that the oPe amplitude tracked with the noticing of model-made errors, and the oLPC tracked with error expectancy as measured by model reliability. We also found correlations between the oLPC amplitude and subjective trust measures. We argue that future work on explainable AI should more closely consider the experimental findings regarding the difficulty people have in incorporating text-based explanations into their model assessment when making recommendations for improving human performance through XAI. We propose that the use of ERPs and other noninvasive psychophysiological measures should be more tightly incorporated into the study of trust in automation to (1) provide continuous, unobtrusive, and multi-dimensional measures of real-time trust in automation and (2) ground the development of future self-report and behavioral scales in quantifiable psychological measures.

## Data Availability

The datasets used and/or analyzed during the current study are available from the corresponding author on reasonable request.
